# KLF4 Induces Mesenchymal–Epithelial Transition (MET) by Suppressing Multiple EMT-Inducing Transcription Factors

**DOI:** 10.3390/cancers13205135

**Published:** 2021-10-13

**Authors:** Ayalur Raghu Subbalakshmi, Sarthak Sahoo, Isabelle McMullen, Aaditya Narayan Saxena, Sudhanva Kalasapura Venugopal, Jason A. Somarelli, Mohit Kumar Jolly

**Affiliations:** 1 Centre for BioSystems Science and Engineering, Indian Institute of Science, Bangalore 560012, India; subbalakshmi@iisc.ac.in (A.R.S.); sarthaksahoo@iisc.ac.in (S.S.); sudhanvakv@iisc.ac.in (S.K.V.); 2 Department of Medicine, Duke University, Durham, NC 27708, USA; isabelle.mcmullen@duke.edu; 3 Department of Biotechnology, Indian Institute of Technology, Kharagpur 721302, India; staradi.saxena@iitkgp.ac.in; 4 Duke Cancer Institute, Duke University, Durham, NC 27708, USA

**Keywords:** KLF4, Mesenchymal–Epithelial Transition (MET), Epithelial–Mesenchymal Plasticity (EMP), epigenetics, mathematical modeling

## Abstract

**Simple Summary:**

Cancer is expected to be the leading cause of death due to noncommunicable diseases in the 21st century. Cancer-related mortality is largely due to metastasis. Cancer cells undergoing metastasis exhibit Epithelial–Mesenchymal Plasticity where they can transition from an epithelial to mesenchymal (EMT) or from a mesenchymal to epithelial (MET) phenotype. These transitions are crucial for the success of various stages of metastasis. Both these processes are modulated by multiple EMT-inducing and MET-inducing factors acting in concert. While EMT inducers are well-recognized, MET inducers are relatively poorly investigated. Here, we investigated the role of KLF4 through mechanism-based mathematical models and transcriptomic data analysis and identified it to be a potential MET inducer by suppressing one or more EMT inducers directly and/or indirectly.

**Abstract:**

Epithelial–Mesenchymal Plasticity (EMP) refers to reversible dynamic processes where cells can transition from epithelial to mesenchymal (EMT) or from mesenchymal to epithelial (MET) phenotypes. Both these processes are modulated by multiple transcription factors acting in concert. While EMT-inducing transcription factors (TFs)—TWIST1/2, ZEB1/2, SNAIL1/2/3, GSC, and FOXC2—are well-characterized, the MET-inducing TFs are relatively poorly understood (OVOL1/2 and GRHL1/2). Here, using mechanism-based mathematical modeling, we show that transcription factor KLF4 can delay the onset of EMT by suppressing multiple EMT-TFs. Our simulations suggest that KLF4 overexpression can promote a phenotypic shift toward a more epithelial state, an observation suggested by the negative correlation of KLF4 with EMT-TFs and with transcriptomic-based EMT scoring metrics in cancer cell lines. We also show that the influence of KLF4 in modulating the EMT dynamics can be strengthened by its ability to inhibit cell-state transitions at the epigenetic level. Thus, KLF4 can inhibit EMT through multiple parallel paths and can act as a putative MET-TF. KLF4 associates with the patient survival metrics across multiple cancers in a context-specific manner, highlighting the complex association of EMP with patient survival.

## 1. Introduction

Cancer is expected to surpass all noncommunicable disease-related deaths in the 21st century, making it a major global public health threat [1]. Nearly all cancer-related deaths can be attributed to the process of metastasis [2]. Metastasizing cells can migrate and invade, traits that enable them to disseminate throughout the body [3]. However, metastasis is a highly inefficient process with attrition rates as high as >99.5% [4]; thus, only a miniscule percentage of metastasizing cells comprise the successful seeding of secondary tumor(s). A key hallmark exhibited by these cells is phenotypic plasticity, i.e., their ability to dynamically switch between phenotypes, empowering them to adapt to the ever-changing microenvironments that they face during metastasis [5,6]. Therefore, it is critical to decode the mechanisms of phenotypic plasticity in order to unravel the dynamics of metastasis and develop therapeutic strategies targeting this insurmountable clinical challenge.

A canonical example of phenotypic plasticity is Epithelial–Mesenchymal Plasticity (EMP), i.e., the bidirectional switching among the epithelial, mesenchymal, and hybrid epithelial/mesenchymal (E/M) phenotypes [7]. Many transcription factors (TFs) capable of inducing an Epithelial–Mesenchymal Transition (EMT) are well-characterized, but those driving the reverse of EMT—a Mesenchymal–Epithelial Transition (MET)—remain relatively poorly investigated. For instance, ZEB1/2, SNAI1/2, TWIST, and GSC (Goosecoid) are EMT-TFs that are often activated by signaling pathways, such as TGFβ, and can drive varying extents of EMT in cancer cells through repressing various epithelial genes (such as E-cadherin) and/or inducing the expression of mesenchymal genes (such as vimentin) [8,9,10,11,12,13]. On the other hand, GRHL1/2 and OVOL1/2 are MET-inducing transcription factors (MET-TFs) that often engage in mutually inhibitory feedback loops with EMT-TFs [14,15,16,17,18]. Recent studies have focused on characterizing the drivers and stabilizers of hybrid E/M phenotypes [19,20,21,22,23], which have been claimed to be the ‘fittest’ for metastasis due to their higher plasticity and tumor initiation potential and ability to drive collective migration [24], manifested as clusters of circulating tumor cells [25]—the primary harbingers of metastasis [26]. The role of hybrid E/M cells in metastasis is supported by clinical studies demonstrating an association of hybrid E/M features with worse clinicopathological traits [27,28,29]. However, to effectively target the hybrid E/M phenotype(s), a better understanding of the emergent dynamics of various coupled intracellular and intercellular regulatory networks involved in partial and/or full EMT/MET is required [30].

Krüppel-like factor 4 (KLF4) is an evolutionarily conserved zinc finger-containing transcription factor [31]. It is associated with terminal differentiation and the homeostasis of various epithelial tissues, including its role in maintaining the stability of adherens junctions and establishing the barrier function of the skin [32,33,34]. It also helps maintain the proliferative and pluripotency properties of embryonic stem cells [35] and is crucial for somatic cell reprogramming [32]. Recently, KLF4 has also been investigated in the context of EMT. For instance, in corneal epithelial homeostasis, KLF4 upregulates the levels of various epithelial markers, such as E-cadherin and claudins, and downregulates mesenchymal markers, such as vimentin and the nuclear localization of β-catenin [36]. KLF4 inhibits EMT in the corneal epithelium by preventing the phosphorylation and nuclear localization of SMAD2, thus attenuating TGF-β signaling [37]. Similarly, in pulmonary fibrosis, KLF4 inhibits TGFβ1-induced EMT in human alveolar epithelial cells [38]. In tumor progression, it has been proposed as both an oncogene and as a tumor suppressor, depending on the context [39,40,41,42]. Thus, a deeper understanding of the roles of KLF4 in tumor progression is needed.

At the molecular level, KLF4 has been shown to inhibit, and be inhibited by, both SNAIL (*SNAI1*) [43,44] and SLUG (*SNAI2*) [45], two of the members of the *SNAI* superfamily that can induce EMT to varying degrees [9,46]. Such a mutually inhibitory feedback loop (also known as a ‘toggle switch’) has also been reported between (a) miR-200 and ZEB1/2 [47], (b) SLUG and SNAIL [48], and (c) SLUG and miR-200 [48]. Thus, KLF4, SNAIL, and SLUG form a ‘toggle triad’ [49]. In addition, KLF4 can self-activate [50], similar to ZEB1 [51], while SNAIL inhibits itself and activates ZEB1/2 [48].

Here, we developed a mechanism-based mathematical model that captures the abovementioned interactions to decode the effects of KLF4 on EMT. Our model predicts that KLF4 can inhibit the progression of EMT by inhibiting the levels of various EMT-TFs; consequently, its overexpression can induce a partial or complete MET, similar to the observations for GRHL2 [52,53,54]. An analysis of in vitro transcriptomic datasets and cancer patient samples from The Cancer Genome Atlas (TCGA) revealed a negative correlation between the KLF4 levels and enrichment of EMT. We also incorporated the impact of the epigenetic influence mediated by KLF4 and SNAIL in a population dynamics scenario and demonstrated that KLF4-mediated ‘epigenetic locking’ can enable resistance to EMT, while SNAIL-mediated effects can drive a stronger EMT. Finally, we propose KLF4 as a potential MET-TF that can repress many EMT-TFs simultaneously and inhibit EMT through multiple parallel paths. These observations are supported by the observed association of KLF4 with patient survival metrics across multiple cancers.

## 2. Results

### 2.1. KLF4 Inhibits the Progression of EMT

**Figure 1:**
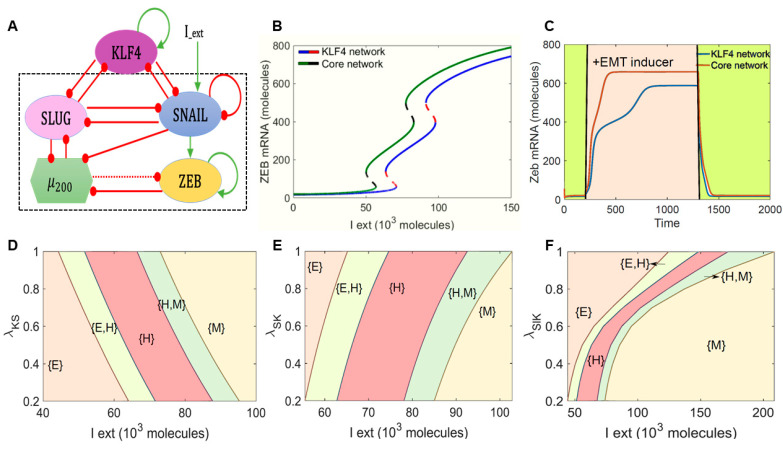
KLF4 inhibits EMT. (**A**) Schematic representation of KLF4 coupled to an EMT regulatory network consisting of miR-200, ZEB1, SNAIL, and SLUG. Green arrows denote activation, and red bars indicate inhibition. Solid arrows represent transcriptional regulation; a dotted line represents microRNA-mediated regulation. The circuit shown within the dotted rectangle is the control case (i.e., core EMT network without KLF4). (**B**) Bifurcation diagrams indicating ZEB1/2 mRNA levels for increasing the external signal (I) levels for the coupled EMT–KLF4 circuit (solid blue and dotted red curve) and the core EMT circuit (solid green and dotted black curve). (**C**) Temporal dynamics of the ZEB1/2 mRNA levels in a cell starting in an epithelial phenotype when exposed to a high level of an external EMT signal (I_ext = 100,000 molecules) (pink-shaded region) for the circuits shown in (**A**)). (**D**–**F**) Phase diagrams for the KLF4–EMT network driven by an external signal (I_ext) for (**D**) varying strengths of repression on SNAIL by KLF4, (**E**) for varying strengths of repression on KLF4 by SNAIL, and (**F**) for varying strengths of repression on SLUG by KLF4.

We began by examining the role of KLF4 in modulating EMT dynamics. To do this we investigated the dynamics of the interaction between KLF4 and a core EMT regulatory circuit (denoted by the black dotted rectangle in Figure 1A) comprised of four players: three EMT-inducing transcription factors (EMT-TFs)—ZEB1/2, SNAIL, and SLUG—and an EMT-inhibiting microRNA family (miR-200).

First, we calculated a bifurcation diagram of ZEB1/2 mRNA levels in response to an external EMT-inducing signal I_ext, which can represent various possible intracellular/extracellular stimuli that can drive EMT (Figure 1B). The ZEB1/2 mRNA levels served as a readout of the EMT phenotypes. We observed that, with an increase in the levels of I_ext, the cells could switch from an epithelial state (ZEB1/2 mRNA < 100 molecules) to a hybrid E/M phenotype (100 < ZEB1/2 mRNA molecules < 600) and, finally, to a mesenchymal state (ZEB1/2 mRNA > 600 molecules). This behavior was observed both in the absence of KLF4 (curve with a green solid line and black dashed line in Figure 1B) and in the presence of KLF4 (curve with a blue solid line and red dashed line in Figure 1B), and the bifurcation curves looked similar in shape. However, in the presence of KLF4, we observed that the system required a stronger EMT signal to be pushed out of an epithelial state and, also, for the acquisition of a completely mesenchymal state (i.e., the bifurcation diagram in the presence of KLF4 was shifted to the right as compared to that of the core network without KLF4). This observation suggests that KLF4 can inhibit the progression of EMT.

To test the robustness of the model prediction for the role of KLF4 in EMT, we performed a sensitivity analysis in which we varied the numerical value of every kinetic parameter used in the model by ±10% one at a time and captured the changes in the range of the I_ext for which the hybrid E/M state existed in the bifurcation diagram. Except for a few parameter cases involving ZEB1/2 and miR200 interactions, this change was found to be less than 10% for a corresponding 10% change in the individual parameter values. Specifically, for variations in the kinetic parameters corresponding to the interactions of KLF4 with the core EMT circuit, this change did not extend beyond 1% (Figure S1A). Thus, the observed behavior of KLF4 in its ability to delay or inhibit EMT is robust to small parametric variations.

Next, we determined the temporal response of cells to a fixed concentration of the external EMT-inducing signal I_ext_. In the absence of KLF4, cells in the epithelial state transitioned first to a hybrid E/M state and then to a mesenchymal state in response to an external signal (red curve in Figure 1C). However, in the presence of KLF4, this transition was much more gradual and relatively slower (blue curve in Figure 1C). In addition, the steady-state value of ZEB1/2 mRNA levels was lower in the presence of KLF4 as compared to the control case. This decrease can be attributed to the KLF4-mediated inhibition of both SLUG and SNAIL that can activate ZEB1/2. Additionally, it was consistent with the trends in ZEB1/2 mRNA level bifurcation diagram (the blue curve lies below the green curve at all the values of I_ext in Figure 1B).

KLF4 inhibits both SLUG and SNAIL and is inhibited by both of them. Thus, we probed the impact of the interactions between KLF4 and both of these EMT-TFs in terms of influencing EMT progression. First, we varied the strength of the repression of SNAIL by KLF4. When this repression was strong (i.e., low λ_KS_ or low K^0^S values), the cells required a stronger EMT-inducing signal to be pushed out of the epithelial state. Conversely, when KLF4 inhibited SNAIL weakly (higher λ_KS_ or K^0^S values), EMT could be induced at lower values of I_ext (Figure 1D and Figure S1B). Next, we varied the repression of KLF4 by SNAIL. At a stronger repression (i.e., low λ_SK_ or low S^0^K values), the cells could exit the epithelial state at a weaker external EMT-inducing signal. Conversely, when SNAIL inhibited KLF4 weakly (higher λ_SK_ or S^0^K values), a stronger stimulus was required for the cells to exit the epithelial state (Figure 1E and Figure S1C). Put together, these results highlighted that, while a weaker impact of KLF4—through either a stronger repression of KLF4 by SNAIL or by a weaker repression of SNAIL by KLF4—potentiated the progression of EMT, a stronger impact of KLF4 prevented cells from undergoing EMT. Similar results were seen for the feedback loop between SLUG and KLF4 (Figure 1F and Figure S1D,E), but the impact on the EMT dynamics was weaker upon altering the inhibition of SLUG by KLF4 than that of SNAIL by KLF4. Upon altering either λ_KSl_ or K^0^Sl, we did not observe any change in concentration of I_ext_ needed to induce EMT, as seen for the case with SNAIL (compare Figure S1D with Figure 1D and Figure S1E with Figure S1B). This difference may be explained by reports suggesting that SNAIL is a more potent EMT inducer than SLUG [9,46]. This hypothesis is strengthened by observations that SLUG self-activation does not alter the qualitative dynamics of the KLF4-SNAIL interactions (Figure S2A–C). A stronger KLF4 self-activation, on the other hand, can increase resistance to undergo EMT (Figure S2D).

### 2.2. KLF4 Promotes an Epithelial Phenotype

We next examined whether the impact of KLF4 in inhibiting EMT can be a generic emergent property of the topology of the regulatory network that it forms with SLUG, SNAIL, and ZEB1/21, instead of the behavior of a specific parameter set. Thus, to map the possible phenotypic space of the network shown in Figure 1A, we simulated its dynamics using the computational framework RACIPE [55]. RACIPE considers the topology of the gene regulatory network as an input and converts that network topology information into a set of coupled ordinary differential equations (ODEs). These ODEs are solved over a wide range of biologically relevant parameter values to identify the different possible steady-state gene expression levels (phenotypes) that the network is capable of giving rise to.

**Figure 2:**
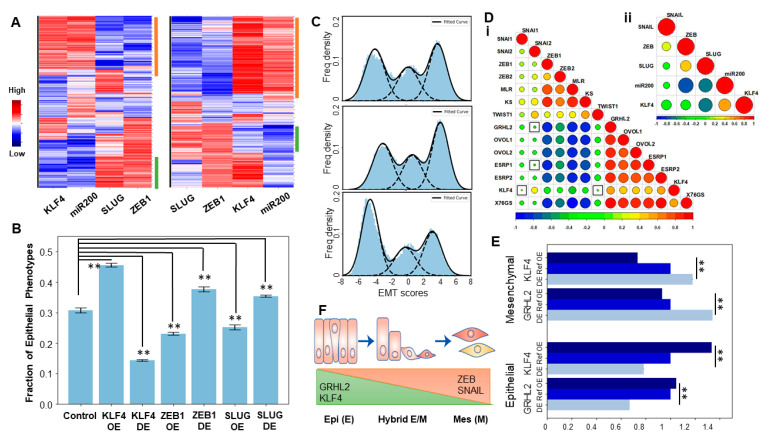
KLF4 promotes an epithelial phenotype. (**A**) (**Left**): Heatmap showing the steady-state values of all the components of the KLF4–EMT-coupled circuit, obtained across the parameter sets simulated by RACIPE. (**Right**) Same as the left panel but for KLF4 overexpression. (**B**) Change in the fraction of the epithelial phenotype due to the simulated overexpression or downregulation of either KLF4 or ZEB1 or SLUG. Error bars for *n* = 3 independent simulations. (**C**) Frequency density of the epithelial, hybrid, and mesenchymal phenotypes obtained using the EMT scores. (**Top**) KLF4 circuit, (**middle**) KLF4 circuit with KLF4 down-expression (DE), and (**bottom**) KLF4 circuit with KLF4 overexpression (OE). (**D**) Pairwise Pearson’s correlation matrix. (**i**) Correlation of the epithelial and mesenchymal players and EMT scoring metrics (76GS, KS, and MLR) in the CCLE cell lines. (**ii**) Correlation of the RACIPE-simulated expression values of the nodes in the KLF4 network. Squares indicate a *p*-value > 0.01. (**E**) Change in the size of the epithelial and mesenchymal clusters upon OE and DE of GRHL2 and KLF4. (**F**) Schematic showing the variations of the KLF4, GRHL2, ZEB1, and SNAIL levels across the EMT spectrum. **: *p* < 0.01.

After obtaining the possible phenotypes from the RACIPE analysis, we plotted the distributions of the steady-state levels of different nodes in the gene regulatory network obtained across the ensemble of parameter sets. KLF4, SLUG, ZEB1, and miR-200 showed bimodal distribution, while SNAIL showed a unimodal distribution (Figure S3A); thus, SNAIL was excluded from the subsequent clustering analysis. We plotted the steady states obtained from RACIPE derived as a heatmap (Figure 2A, left). Hierarchical clustering performed on the heatmap revealed two predominant clusters—one with low ZEB1/2, low SLUG, high miR200, and high KLF4 and another with high ZEB1/2, high SLUG, low miR200, and low KLF4 levels. These clusters can be mapped onto epithelial and mesenchymal phenotypes, respectively (green and orange bars in Figure 2A). Next, we perturbed the production rates of KLF4 to mimic the over- and under-expression of KLF4 and assessed its effect on the frequency of the observed epithelial and mesenchymal phenotypes. Overexpression of KLF4 led to an increased frequency of the epithelial cluster and a decrease in the mesenchymal cluster (Figure 2A, right). As expected, opposite patterns were observed upon the inhibition and over-expression of ZEB1, an EMT-TF (Figure S3B). We quantified the change in the fraction of the epithelial phenotype when KLF4, ZEB1, and SLUG are either overexpressed or inhibited one at a time. The overexpression of KLF4 or downregulation of either SLUG or ZEB1 increased the frequency of the epithelial phenotype (Figure 2B).

To investigate the classification of phenotypes in a more quantitative manner, and to characterize heterogeneity in EMT in a given cell population, we defined an EMT score (= ZEB1 + SLUG − miR-200 − CDH1) for an extended regulatory network that included E-cadherin and its connections with SLUG and ZEB1 (Figure S3C). The distribution of the EMT scores plotted for the solutions seen across the parameter sets in RACIPE revealed a trimodal distribution, indicating the existence of three distinct EMT phenotypes: epithelial, mesenchymal, and hybrid E/M (Figure 2C, top panel). A simulated knockdown of KLF4 drove an increase in the mesenchymal subpopulation with a concurrent decrease in the epithelial states (Figure 2C, middle panel). Opposite trends were observed for the case of simulated KLF4 overexpression (Figure 2C, bottom panel). These results highlighted that the emergent dynamics of the KLF4–EMT network can allow for multiple phenotypes to coexist in an isogenic population; and the KLF4 levels can modulate the distribution of phenotypic heterogeneity in that population towards a more epithelial or a more mesenchymal state.

To interrogate the role of KLF4 in EMT/MET further, we performed a pairwise correlation analysis in the Cancer Cell Line Encyclopedia (CCLE) cohort among three distinct transcriptomic-based EMT scoring metrics (76GS, KS, and MLR) and the expression levels of KLF4 and those of the other canonical epithelial and mesenchymal factors. KLF4 correlated negatively with the KS and MLR EMT scoring metrics (higher KS or MLR scores denote a mesenchymal phenotype [56]) but positively with the 76GS scores (higher 76GS scores denote a more epithelial phenotype [56]) (Figure 2D,i). Most EMT-TFs were found to be correlated positively with each other (SNAI1/2, ZEB1/2, and TWIST1) and negatively with KLF4 and the other MET drivers, such as ESRP1/2, OVOL1/2, and GRHL2 [57], which were all positively corelated with KLF4 (Figure 2D,i). Consistent correlations were recapitulated in the RACIPE simulation data for the KLF4–EMT network (Figure 2D,ii), thus underscoring that the gene regulatory network considered in Figure 1A can explain these observed experimental trends for the existence of ‘teams’ [58] of EMT and MET inducers. Interestingly, GRHL2 seemed to correlate more strongly with ZEB1, ZEB2, and TWIST1 and the MLR and KS scores as compared to KLF4 (Figure 2D,i), thus encouraging us to compare the influence of KLF4 and GRHL2 in terms of their ability to induce MET via simulations. We compared the over expression (OE) and down expression (DE) scenarios of GRHL2 and KLF4 in terms of influencing the distribution of the epithelial and mesenchymal phenotypes and noted a stronger enrichment of mesenchymal upon the downregulation of GRHL2 than that seen upon the downregulation of KLF4 (Figure 2E and Figure S3D). Thus, our results suggest that KLF4, similar to GRHL2, can induce a partial or full MET (Figure 2F).

### 2.3. KLF4 Is Inhibited during EMT

**Figure 3:**
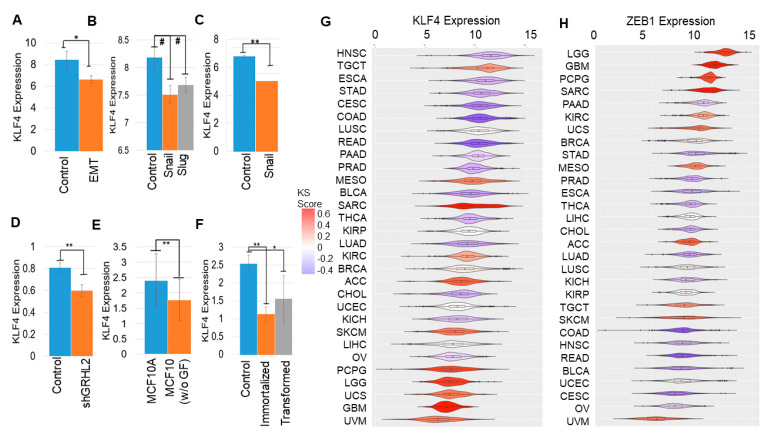
KLF4 is inhibited during EMT. KLF4 expression across comparison groups in GEO datasets. (**A**) GSE59922, (**B**) GSE40690, (**C**) GSE58252, (**D**) GSE118407, (**E**) GSE85857, and (**F**) GSE110677. #: *p* < 0.1, *: *p* < 0.05, and **: *p* < 0.01 for a Student’s two-tailed *t*-test with unequal variances. (**G**,**H**) KLF4 and ZEB1 expression in TCGA types, arranged by mean KS scores (color scheme given on the right).

Next, using various publicly available transcriptomic datasets, we examined if KLF4 is inhibited as cells undergo EMT. In mouse mammary cells EpRas induced to undergo EMT by TGFβ treatment for 14 days [59], KLF4 levels were reduced (GSE59922; Figure 3A). Similarly, when EMT was induced in HMEC cells via the overexpression of SNAIL or SLUG [9], KLF4 levels went down (GSE40690; Figure 3B). Reinforcing trends were seen in MCF-7 cells forced to undergo EMT via the overexpression of SNAIL [60] (GSE58252; Figure 3C), in OVCA4209 cells in which GRHL2 was knocked down [61] (GSE118407; Figure 3D), and in MCF10A cells cultured without growth factors and shown to undergo EMT [62] (GSE85857; Figure 3E). Further, as compared to the primary HMEC (human mammary epithelial cells), immortalized and Ras-transformed HMECs were enriched for EMT-associated genes (GSE110677) and had lower KLF4 levels (Figure 3F). Together, these datasets across cancer types reveal a robust reduction in KLF4 expression with the onset of EMT.

To substantiate these in vitro observations with clinical data, we compared KLF4 levels across cancers in The Cancer Genome Atlas (TCGA). KLF4 expression was found to be lower in cancers with a more mesenchymal phenotype, as measured by their higher KS-based EMT scores [63]. Mesenchymal-like cancers, such as uveal melanoma (UVM), uterine carcinosarcoma (UCS), glioblastoma (GBM), and low-grade glioma (LGG), tend to have lower KLF4 expressions (shown in red in Figure 3G). Conversely, the KLF4 levels were higher in epithelial-like cancer types, such as the head and neck squamous cell carcinoma (HNSC), esophageal carcinoma (ESCA), stomach adenocarcinoma (STAD), and cervical carcinoma (CESC) samples (shown in blue; Figure 3G). ZEB1 and SNAIL, on the other hand, showed opposite trends to KLF4: enriched in cancers with a higher KS score: LGG, GBM, UCS, SARC (sarcoma), and PCPG (pheochromocytoma and paraganglioma) but reduced in those with a lower one: HNSC, COAD (colorectal adeno-carcinoma), CESC, BLCA (bladder carcinoma), and READ (rectum adenocarcinoma) (Figure 3H and Figure S4A). Hence, an inverse correlation of KLF4 with multiple EMT-TFs seen in vitro is consistently observed in TCGA samples.

### 2.4. Epigenetic Changes, including KLF4 Promoter Methylation, Can Alter Population Distributions along the EMT Spectrum

**Figure 4:**
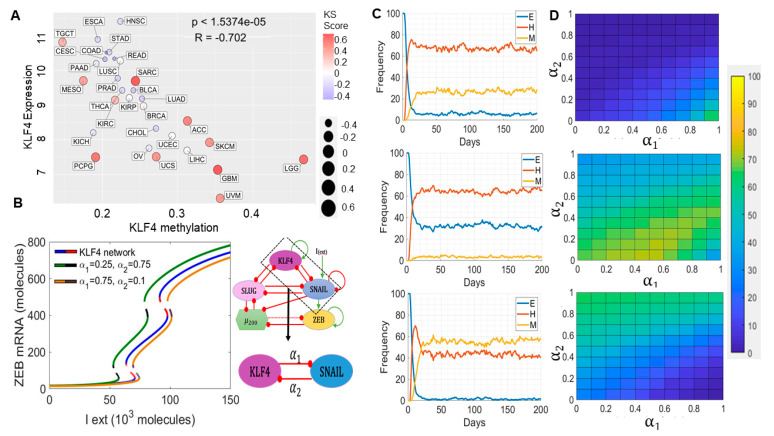
Epigenetic modulations involving KLF4 can alter the population dynamics of EMT states. (**A**) Scatter plot for KLF4 expression and its methylation status in TCGA types. (**B**) Bifurcation diagrams indicating the ZEB mRNA levels for increasing the EMT-inducing external signal (I_ext) levels for the coupled EMT–KLF4 circuit (solid blue and dotted red curve), for the circuit with higher α_1_ and lower α_2_ values (solid yellow and dotted brown curve), and for the circuit with lower α_1_ and higher α_2_ values (solid green and dotted black curve). (**C**) Stochastic simulation of the KLF4–EMT network for varied values of α_1_ and α_2_. (**Top**) α_1_ = α_2_ = 0, (**middle**) α_1_ = 0.75 and α_2_ = 0.1, and (**bottom**) α_1_ = 0.25 and α_2_ = 0.75. (**D**) Population distribution of E (**top**), hybrid E/M (**middle**), and M (**bottom**) cells for varying values of α_1_ and α_2_. In panel A; 1.5374e-05 means 1.5374 × 10^−5^.

A decrease in KLF4 expression has been reported to be associated with the hypermethylation of the KLF4 promoter during EMT in renal fibrosis in vitro and in vivo [64]. Thus, we examined the correlation of KLF4 expression with its methylation status in TCGA data. We observed a reduced methylation of KLF4 in many cancers with reduced KS scores, such as HNSC, ESCA, and COAD. Consistent with this observation, KLF4 expression and methylation status were negatively correlated (Figure 4A), reminiscent of the observations in the renal cancer cell lines and tissues and suggesting a possible epigenetic mechanism driving its suppression during EMT. Consistently, a DNA methyltransferase inhibitor increased KLF4 expression in renal cancers [65]. SNAIL expression was also negatively correlated with the corresponding promoter methylation levels in TCGA; however, ZEB1 did not show a clear pattern (Figure S4B,C). These observations drove us to investigate the impact of the epigenetic influence operating in the KLF4 and SNAIL feedback loop.

Epigenetic changes can drastically alter the rates of transition among the different cell phenotypes by controlling the accessibility of the promoters for ‘master regulators’. In the context of EMT, we have previously shown that epigenetic feedback mediated by ZEB1 while repressing miR-200 (i.e., blocking the access of EMT inducers to the miR-200 promoter) can drive irreversible EMT, while that mediated by GRHL2 (i.e., blocking access to the ZEB1 promoter for EMT inducers) in inhibiting ZEB1 can enable irreversible MET, i.e., a resistance of cells to undergo EMT [66,67]. Here, we assessed the impact of the KLF4-mediated epigenetic silencing of SNAIL (i.e., the ability of KLF4 to cause methylation of the SNAIL promoter directly or indirectly) and vice versa (SNAIL-mediated epigenetic silencing of KLF4) with a population dynamics model capturing a cell population with diverse EMT states (epithelial, mesenchymal, and hybrid E/M). This phenomenological model encapsulates the epigenetic influence by modulating the threshold for the impact of a transcription factor on the expression of its downstream target [68]. Such dynamic thresholds capturing the epigenetic influence often enable the self-stabilization of gene expression states, i.e., the longer a transcription factor has been active, the easier it becomes for it to stay ‘on’. We introduced two epigenetic variables: α_1_ and α_2_. The higher the value of α_1_, the stronger is the influence of the KLF4-mediated effective epigenetic silencing of SNAIL. The higher the value of α_2_, the stronger is the influence of the SNAIL-mediated effective epigenetic silencing of KLF4 (see Methods for details).

As a first step towards understanding the dynamics of this epigenetic ‘tug of war’ between KLF4 and SNAIL, we characterized how the bifurcation diagram of the KLF4–EMT-coupled circuit changed at various values of α_1_ and α_2_. When the epigenetic silencing of SNAIL mediated by KLF4 was higher than that of KLF4 mediated by SNAIL ((α_1_, α_2_) = (0.75, 0.1)), a larger EMT-inducing signal (I_ext) was required to push cells out of an epithelial state, because SNAIL was being strongly repressed by KLF4 as compared to the control case in which there is no epigenetic influence (compare the blue/red curve with the black/yellow curve in Figure 4B). Conversely, when the epigenetic silencing of KLF4 predominated ((α_1_, α_2_) = (0.25, 0.75)), it was easier for cells to exit an epithelial state, presumably because the KLF4 repression of EMT was now being inhibited more potently by SNAIL relative to the control case (compare the blue/red curve with the black/green curve in Figure 4B). Thus, these opposing epigenetic ‘forces’ can ‘push’ the bifurcation diagram in different directions along the *x*-axis without impacting any of its major qualitative features.

To consolidate these results, we next performed stochastic simulations for a population of 500 cells at a fixed value of I_ext = 90,000 molecules. We observed a stable phenotypic distribution with 6% epithelial (E), 28% mesenchymal (M), and 66% hybrid E/M cells (Figure 4C, top) in the absence of any epigenetic regulation (α_1_ = α_2_ = 0). In the case of a stronger epigenetic repression of SNAIL by KLF4 (α_1_ = 0.75, α_2_ = 0.1), the population distribution changed to 32% epithelial (E), 3% mesenchymal (M), and 65% hybrid E/M cells (Figure 4C, middle). Conversely, when SNAIL repressed KLF4 more dominantly (α_1_ = 0.25 and α_2_ = 0.75), the population distribution changed to 1% epithelial (E), 58% mesenchymal (M), and 41% hybrid E/M cells (Figure 4C, bottom). A similar analysis was performed for collating steady-state distributions for a range of α_1_ and α_2_ values, revealing that high α_1_ and low α_2_ values favored the predominance of an epithelial phenotype (Figure 4D, top), but low α_1_ and high α_2_ values facilitated a mesenchymal phenotype (Figure 4D, bottom). Intriguingly, when the strength of the epigenetic repression from KLF4 to SNAIL and vice versa was comparable, the hybrid E/M phenotype dominated (Figure 4D, middle). Put together, varying extents of epigenetic silencing mediated by EMT-TF SNAIL and a MET-TF KLF4 can fine tune the epithelial–hybrid-mesenchymal heterogeneity patterns in a cell population.

### 2.5. KLF4 Correlates with Patient Survival

**Figure 5:**
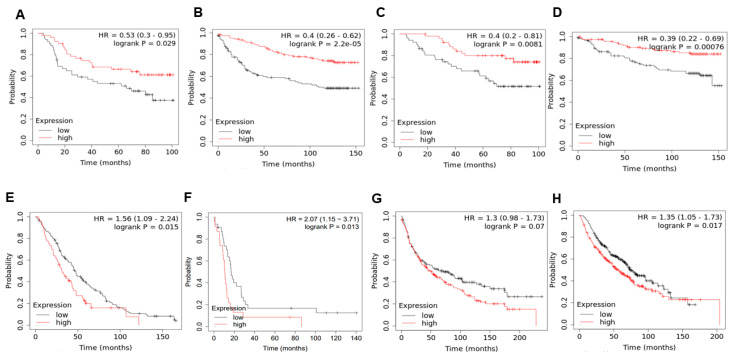
KLF4 correlates with patient survival in a cancer-specific manner. (**A**,**B**) Relapse-free survival trends in GSE42568 and GSE3494 (breast cancer), respectively. (**C**,**D**) Same as (**A**,**B**) but for the overall survival. (**E**,**F**) Overall survival trends in GSE26712 and GSE30161 (ovarian cancer), respectively. (**G**,**H**) Overall survival trends in GSE30219 and CaArray (lung cancer), respectively. HR denotes the hazard ratio, and logrank P denotes the *p*-value. The mean value and 95% confidence interval are shown. In panel B; 2.2e-05 means 2.2 × 10^−5^.

To determine the effects of KLF4 on clinical outcomes, we investigated the correlation between KLF4 and patient survival. We observed that high KLF4 levels correlated with better relapse-free survival (Figure 5A,B) and better overall survival (Figure 5C,D) in two specific breast cancer datasets—GSE42568 (*n* = 104 breast cancer biopsies) [69] and GSE3494 (*n* = 251 primary breast tumors) [70]. However, the trend was reversed in terms of the overall survival data (Figure 5E,F) in ovarian cancer—GSE26712 (*n* = 195 tumor specimens) [71] and GSE30161 (*n* = 58 cancer samples) [72] and lung cancer (Figure 5G,H)—GSE30219 (*n* = 293 lung tumor samples) [73] and caArray (*n* = 504 samples) [74], where high KLF4 levels correlated with worse patient outcomes.

Given that high KLF4 expression associates with a more epithelial phenotype, these results, when extrapolated to indicate the extent of EMT/MET, suggest that EMT associates with a worse survival in breast cancer but not necessarily in ovarian cancer and lung cancer, as far as these limited datasets being analyzed are concerned. These results are reminiscent of previous observations that EMT need not universally correlate with worse patient survival outcomes and can depend on the cancer type being investigated [63,75]. Therefore, the association of KLF4 with survival seems to be tumor type-specific, and future studies are needed to decipher the interplay between KLF4 and EMT/MET states as a prognostic marker of clinical outcomes in a cancer-specific manner.

## 3. Discussion

We hereby propose KLF4 as a potential MET-inducing transcription factor (MET-TFs) based on in silico model predictions and their experimental validation across multiple in vitro and cancer patient sample datasets. This observation adds to the increasing literature on the role of KLF4 in inhibiting EMT and/or driving MET in different biological contexts. For instance, in the colon epithelial cell line RKO, KLF4 upregulates the levels of various epithelial-specific keratins, such as KRT8 and KRT18 [76]. Similarly, in nasopharyngeal carcinoma, KLF4 can transcriptionally activate E-cadherin and reduce the motility and invasion of cells. This reduction is at least partly rescued by shRNA-mediated E-cadherin knockdown in KLF4-expressing cells, suggesting a functional role of E-cadherin in regulating these traits [77]. The direct transcriptional activation of CDH1 (E-cadherin) by KLF4 has also been noted in MCF10A cells [78]. Further, the overexpression of KLF4 in MDA-MB-231 breast cancer cells restored E-cadherin levels, induced an epithelial morphology, and suppressed migration and invasion [78], similar to previous observations in these cells for another MET-TF, GRHL2 [14]. Consistently, KLF4 overexpression decreased levels of vimentin and Slug and increased those of E-cadherin in OVCAR3 ovarian cancer cells [79]. These observations are reminiscent of the effect of KLF4 knockdown in a prostate stem cell line where the cells lost their epithelial markers, such as E-cadherin, ZO-1, and cytokeratin 8, and showed elevated levels of vimentin, SNAIL, SLUG, and ZEB1 [80]. Supporting these in vitro observations, in pancreatic cancer samples, KLF4 correlated positively with E-cadherin and negatively with vimentin and Cav-1, a direct transcriptional target of KLF4 that can inhibit EMT in pancreatic cancer [81].

KLF4 can also promote stemness in various cancers where it promotes epithelial differentiation, thereby challenging the tacitly assumed association between EMT and cancer stem cells (CSCs) [82]. In breast cancer, KLF4 knockdown reduced ALDH1^+^ CSCs and mammosphere formation in vitro in MCF7 and MDA-MB-231 cells [41]. In vivo tumorigenesis and metastasis were also compromised in KLF4-depleted NOD/SCID mice [41,83]. In hepatocellular carcinoma, KLF4 directly activated EpCAM, increased the number of EpCAM^+^/CD133^+^ liver cancer stem cells in vitro, and amplified the tumorigenesis in vivo [84]. Similarly, in osteosarcoma cells, KLF4 suppression prevented sphere formation and attenuated the levels of many stem cell-related markers, including ALDH1A1 [85]. Conversely, KLF4-overexpressing cells were more chemoresistant and metastatic [86], and osteosarcoma stem cells had increased levels of KLF4 [87]. Considered together, these observations suggest that KLF4 may associate with more epithelial-like and/or hybrid E/M CSC phenotypes [88], similar to NRF2 [89]. Moreover, KLF4 can have additional cellular functions too, such as cell cycle regulation and metabolic reprogramming [90,91]. Thus, a more comprehensive analysis of the role of KLF4 on various axes of cellular plasticity is needed.

However, the association of KLF4 with metastasis is relatively ambiguous and context dependent. High KLF4 was shown to prevent metastasis in breast cancer [92] and pancreatic cancer models [93]. KLF4 can also have additional roles in modulating metastasis beyond regulating EMT and/or stemness, such as in its pro-metastasis role in the phenotypic switching of perivascular cells and formation of a premetastatic niche [94]. Another potential confounding factor is the association of KLF4 with cell cycle regulation. KLF4 can drive cell cycle arrest [76], but it also simultaneously represses p53 and activate p21^CIP^ [42], acting as an ‘incoherent signal’ that can drive antagonistic outputs depending on the relative strengths of the regulations. Therefore, future studies investigating the coupled dynamics of EMT, stemness, cell cycle, and the connection of KLF4 to these regulatory pathways are needed to elucidate the impact of KLF4 in modulating these interconnected axes driving metastasis. Additionally, further developments of small-molecule inhibitors or inducers of KLF4 [95,96] will be helpful in identifying the protumor or antitumor roles of KLF4 in patients.

Overall, our analysis highlights KLF4 as a potential MET-TF, adding to the list of known MET-TFs, such as GRHL1/2/3 and OVOL1/2. Recent studies showing that MET is not simply the inverse of EMT [12,97,98] necessitate attention to identify more MET-TFs and the interconnected networks they form with EMT-TFs to gain a comprehensive understanding of the emergent dynamics of epithelial-mesenchymal plasticity in metastatic–invasion cascade.

## 4. Materials and Methods

### 4.1. Mathematical Modeling

As per the schematic shown in Figure 1A, the dynamics of all the four molecular species (miR-200, SNAIL, ZEB, and SLUG) were described by a system of coupled ODEs. The generic chemical rate equation given below describes the level of a protein, mRNA, or microRNA (*X*):

(1) $$\frac{dX}{dt}=g_{X}H^{S}\left(A,A_{0},n,\lambda \right)-k_{X}X$$

where the first term *g_X_* signifies the basal rate of production, and the terms multiplied to *g_X_* represent transcriptional/translational/post-translational regulations due to interactions among the species in the system, as defined by the shifted Hills function ($H^{S}\left(A,A_{0},n,\lambda \right)$). The rate of degradation of the species (*X*) is defined by the term *k_X_X* based on first-order kinetics. The complete set of equations and parameters are presented in the File S1. The bifurcation diagrams were drawn using the continuation software package MATCONT [99].

### 4.2. RACIPE (Random Network Simulation)

The gene regulatory network shown in Figure 1A was simulated via RACIPE. The overexpression and down-expression of KLF4, ZEB1, and SLUG were done by setting the fold change value to 10. Ten thousand parameter sets were simulated for 100 different initial conditions to obtain the ensemble of steady-state solutions. The steady-state solutions were Z-normalized for each gene over all the steady-state values as the observed steady-state expression—mean steady-state expression)/standard deviation of the steady-state expression. The resultant normalized steady-state solutions were plotted as a heatmap. Significance in the differences between distinct groups were accessed by performing a Students *t*-test on three replicates of 10,000 parameter sets each.

Next, we incorporated CDH1 to the circuit in Figure 1A and simulated the GRN by RACIPE. A similar circuit was also simulated by incorporating GRHL2 but without KLF4. Along with the base circuits, the overexpression and down-expression were also done for KLF4 and GRHL2 50-fold in their respective circuits. The RACIPE steady states were z-normalized as above, and the EMT score for each steady state was calculated as ZEB1 + SLUG − miR-200 − CDH1. The resultant trimodal distribution was quantified by fitting 3 gaussians. The frequencies of the epithelial and mesenchymal phenotypes were quantified by computing the area under the corresponding gaussian fits. Significance in the difference between the distinct groups was accessed by performing a Students’ *t*-test on three replicates of 10,000 parameter sets each.

### 4.3. Gene Expression Datasets

The gene expression datasets were downloaded using the GEOquery R Bioconductor package [100]. Preprocessing of these datasets was performed for each sample to obtain the gene-wise expression from the probe-wise expression matrix using R (version 4.0.0).

### 4.4. External Signal Noise and Epigenetic Feedback on KLF4 and SNAIL

The external noise and epigenetic feedback calculations were performed as described earlier [67].

- Noise on External signal:

The external signal I that we use here can be written as the stochastic differential equation:

$$\dot{I}=\beta \left(I_{0}-I\right)+\eta \left(t\right)$$

where η(t) satisfies the condition $\eta \left(t\right),\;n\left(t^{\prime }\right)\geq N\delta \left(t-t^{\prime }\right)$. Here, *I*_0_ is set at 90-K molecules, β as 0.04 h-1, and N as 80-K molecules/hour^2^.

- Epigenetic feedback:

We tested the epigenetic feedback on the KLF4-SNAIL axis. The dynamic equation of epigenetic feedback on the inhibition by KLF4 on SNAIL is:

$$\dot{K_{S}^{0}=}\frac{K_{S}^{0}\left(0\right)-K_{S}^{0}-\alpha K}{\zeta }$$

Similarly, the epigenetic feedback on the SNAIL inhibition on KLF4 is modeled via:

$$\dot{S_{K}^{0}=}\frac{S_{K}^{0}\left(0\right)-S_{K}^{0}-\alpha S}{\zeta }$$

where *ζ* is a timescale factor and chosen to be 100 (hours). *α* represents the strength of epigenetic feedback. A larger α corresponds to stronger epigenetic feedback. *α* has an upper bound because of the restriction that the numbers of all the molecules must be positive. For inhibition by KLF4 on SNAIL, a high level of KLF4 can inhibit the expression of SNAIL due to this epigenetic regulation. Meanwhile, for SNAIL’s inhibition on KLF4, high levels of SNAIL can suppress the synthesis of KLF4.

### 4.5. Kaplan-Meier Analysis

KM Plotter [74] was used to conduct the Kaplan–Meier analysis for the respective datasets. The number of samples in the KLF4-high vs. KLF4-low categories is given in File S1.

### 4.6. Patient Data

The gene expression levels for the batch effect normalized RNA-seq were obtained for 12,839 samples from The Cancer Genome Atlas’s (TCGA) pan-cancer (PANCAN) dataset via the University of California, Santa Cruz’s Xena Browser. The samples were filtered using unique patient identifiers, and only samples that overlapped between the two datasets were kept (11,252 samples). The samples were further filtered to remove patients with missing data for the gene expression or cancer type (10,619 samples). These samples were ultimately used in all the subsequent analyses. The DNA methylation data were obtained from the TCGA PANCAN dataset via the University of California, Santa Cruz’s Xena Browser. The methylation data were profiled using the Illumina Infinium HumanMethylation450 Bead Chip (450K) [101].

### 4.7. EMT Score

The Epithelial–Mesenchymal Transition (EMT) score was calculated using the Kolmogorov–Smirnov (KS) scoring metric [63]. For a given patient, the cumulative distribution functions (CDFs) of the Epithelial and Mesenchymal gene signatures were compared. First, the distance between Epithelial and Mesenchymal signatures was calculated using the maximum distance between their cumulative distribution functions. This represented the test statistic in the subsequent two-sample test used to calculate the EMT score. The score was ultimately determined by the hypothesis testing of two alternative hypotheses, with the null hypothesis being that there was no difference in the CDF of the Epithelial and Mesenchymal signatures. The first hypothesis was that the CDF of the Mesenchymal signature was greater than the CDF of the Epithelial signature. The second hypothesis was that the CDF of the Epithelial signature was greater than the CDF of the Mesenchymal signature. This scoring metric ranged from −1 to +1, where a sample with a positive EMT score was Mesenchymal, whereas negative EMT scores were associated with an Epithelial phenotype. The MLR and 76GS scores were calculated as reported earlier [56,102].

### 4.8. Methylation Status

After the four genes of interest were identified as KLF4, SLUG (SNAI2), SNAIL (SNAI1), and ZEB1, the methylation and expression data were obtained from the TCGA PANCAN dataset via the University of California, Santa Cruz’s Xena Browser. The methylation data was quantified from the Illumina Infinium HumanMethylation450 Bead Chip (450K) for each of the four genes and identified the unique CpG associated with each of these genes: 10 unique CpG sites for KLF4, 10 for SLUG, 13 for SNAIL, and 42 for ZEB1. A β-value representing how methylated each CpG site was, with a β-value of one representing a fully methylated CpG site, was provided for all patients and at every CpG site. To quantify the methylation status of each gene, the β-values for all the associated CpG sites were averaged [101]. The gene expression was then visualized by using R’s ggplot2 package to display violin plots for each gene that was ordered by the gene expression and colored by the EMT score. Subsequently, the methylation data and expression data were again plotted using R’s ggplot2 package, with labels created via R’s ggrepel package.

## 5. Conclusions

Our study indicates KLF4 as a potential MET-inducing transcription factor by suppressing multiple EMT transcription factors directly or indirectly—SNAIL, SLUG, and ZEB1. The simulations for our mechanistic model indicated a delay in the onset of EMT in the presence of KLF4. Further, KLF4 was found to be negatively correlated with EMT and EMT-TFs in multiple transcriptomic datasets. Finally, the epigenetic influence mediated by KLF4 on EMT can change the phenotypic distribution in a heterogeneous cancer cell population. Thus, KLF4 can inhibit EMT through multiple parallel paths and can act as a putative MET-TF.

## Data Availability

The data presented in this study are available in this article (and the supplementary materials). All codes used in the manuscript are available on GitHub at https://github.com/ARShuba/KLF4, accessed on 27 August 2021).

## Supplementary Materials

The following are available online at https://www.mdpi.com/article/10.3390/cancers13205135/s1: Figure S1: Effect of KLF4 on EMT, Figure S2: Effect of SLUG self-activation, Figure S3: RACIPE analysis of KLF4-EMT circuit, Figure S4: EMT score and methylation of TCGA cancers, File S1: KLF4_SI.

## References

[1] Bray F., Ferlay J., Soerjomataram I., Siegel R.L., Torre L.A., Jemal A. (2018). Global cancer statistics 2018: GLOBOCAN estimates of incidence and mortality worldwide for 36 cancers in 185 countries. CA. Cancer J. Clin..

[2] Gupta G.P., Massagué J. (2006). Cancer metastasis: Building a framework. Cell.

[3] Chaffer C.L., Weinberg R.A. (2011). A perspective on cancer cell metastasis. Science.

[4] Celià-Terrassa T., Jolly M.K. (2020). Cancer Stem Cells and Epithelial-to-Mesenchymal Transition in Cancer Metastasis. Cold Spring Harb. Perspect. Med..

[5] Welch D.R., Hurst D.R. (2019). Defining the Hallmarks of Metastasis. Cancer Res..

[6] Jolly M.K., Celià-Terrassa T. (2019). Dynamics of Phenotypic Heterogeneity Associated with EMT and Stemness during Cancer Progression. J. Clin. Med..

[7] Nieto M.A., Huang R.Y., Jackson R.A., Thiery J.P. (2016). EMT: 2016. Cell.

[8] Drápela S., Bouchal J., Jolly M.K., Culig Z., Souček K. (2020). ZEB1: A Critical Regulator of Cell Plasticity, DNA Damage Response, and Therapy Resistance. Front. Mol. Biosci..

[9] Gras B., Jacqueroud L., Wierinckx A., Lamblot C., Fauvet F., Lachuer J., Puisieux A., Ansieau S. (2014). Snail family members unequally trigger EMT and thereby differ in their ability to promote the neoplastic transformation of mammary epithelial cells. PLoS ONE.

[10] Burk U., Schubert J., Wellner U., Schmalhofer O., Vincan E., Spaderna S., Brabletz T. (2008). A reciprocal repression between ZEB1 and members of the miR-200 family promotes EMT and invasion in cancer cells. EMBO Rep..

[11] Cook D.P., Vanderhyden B.C. (2020). Context specificity of the EMT transcriptional response. Nat. Commun..

[12] Celià-Terrassa T., Bastian C., Liu D.D., Ell B., Aiello N.M., Wei Y., Zamalloa J., Blanco A.M., Hang X., Kunisky D. (2018). Hysteresis control of epithelial-mesenchymal transition dynamics conveys a distinct program with enhanced metastatic ability. Nat. Commun..

[13] Taube J.H., Herschkowitz J.I., Komurov K., Zhou A.Y., Gupta S., Yang J., Hartwell K., Onder T.T., Gupta P.B., Evans K.W. (2010). Core epithelial-to-mesenchymal transition interactome gene-expression signature is associated with claudin-low and metaplastic breast cancer subtypes. Proc. Natl. Acad. Sci. USA.

[14] Somarelli J.A., Shetler S., Jolly M.K., Wang X., Bartholf Dewitt S., Hish A.J., Gilja S., Eward W.C., Ware K.E., Levine H. (2016). Mesenchymal-epithelial transition in sarcomas is controlled by the combinatorial expression of microRNA 200s and GRHL2. Mol. Cell. Biol..

[15] Mooney S.M., Talebian V., Jolly M.K., Jia D., Gromala M., Levine H., McConkey B.J. (2017). The GRHL2/ZEB Feedback Loop-A Key Axis in the Regulation of EMT in Breast Cancer. J. Cell. Biochem..

[16] Saxena K., Srikrishnan S., Celia-Terrassa T., Jolly M.K. (2020). OVOL1/2: Drivers of Epithelial Differentiation in Development, Disease, and Reprogramming. Cells Tissues Organs.

[17] Frisch S.M., Farris J.C., Pifer P.M. (2017). Roles of Grainyhead-like transcription factors in cancer. Oncogene.

[18] Sundararajan V., Pang Q.Y., Choolani M., Huang R.Y.-J. (2020). Spotlight on the Granules (Grainyhead-Like Proteins)—From an Evolutionary Conserved Controller of Epithelial Trait to Pioneering the Chromatin Landscape. Front. Mol. Biosci..

[19] Subbalakshmi A.R., Kundnani D., Biswas K., Ghosh A., Hanash S.M., Tripathi S.C., Jolly M.K. (2020). NFATc Acts as a Non-Canonical Phenotypic Stability Factor for a Hybrid Epithelial/Mesenchymal Phenotype. Front. Oncol..

[20] Pastushenko I., Mauri F., Song Y., de Cock F., Meeusen B., Swedlund B., Impens F., Van Haver D., Opitz M., Thery M. (2021). Fat1 deletion promotes hybrid EMT state, tumour stemness and metastasis. Nature.

[21] Selvaggio G., Canato S., Pawar A., Monteiro P.T., Guerreiro P.S., Brás M.M., Janody F., Chaouiya C. (2020). Hybrid Epithelial-Mesenchymal Phenotypes Are Controlled by Microenvironmental Factors. Cancer Res..

[22] Bui N.H.B., Napoli M., Davis A.J., Abbas H.A., Rajapakshe K., Coarfa C., Flores E.R. (2020). Spatiotemporal regulation of ∆Np63 by TGFβ-regulated miRNAs is essential for cancer metastasis. Cancer Res..

[23] Bocci F., Tripathi S.C., Vilchez Mercedes S.A., George J.T., Casabar J.P., Wong P.K., Hanash S.M., Levine H., Onuchic J.N., Jolly M.K. (2019). NRF2 activates a partial epithelial-mesenchymal transition and is maximally present in a hybrid epithelial/mesenchymal phenotype. Integr. Biol..

[24] Campbell K., Rossi F., Adams J., Pitsidianaki I., Barriga F.M., Garcia-Gerique L., Batlle E., Casanova J., Casali A. (2019). Collective cell migration and metastases induced by an epithelial-to-mesenchymal transition in Drosophila intestinal tumors. Nat. Commun..

[25] Jolly M.K., Mani S.A., Levine H. (2018). Hybrid epithelial/mesenchymal phenotype(s): The ‘fittest’ for metastasis?. Biochim. Biophys. Acta-Rev. Cancer.

[26] Aceto N., Bardia A., Miyamoto D.T., Donaldson M.C., Wittner B.S., Spencer J.A., Yu M., Pely A., Engstrom A., Zhu H. (2014). Circulating tumor cell clusters are oligoclonal precursors of breast cancer metastasis. Cell.

[27] Godin L., Balsat C., Van Eycke Y., Allard J., Royer C., Remmelink M., Pastushenko I., Haene N.D., Blanpain C., Salmon I. (2020). A Novel Approach for Quantifying Cancer Cells Showing Hybrid Epithelial/Mesenchymal States in Large Series of Tissue Samples: Towards a New Prognostic Marker. Cancers.

[28] Jolly M.K., Somarelli J.A., Sheth M., Biddle A., Tripathi S.C., Armstrong A.J., Hanash S.M., Bapat S.A., Rangarajan A., Levine H. (2019). Hybrid epithelial/mesenchymal phenotypes promote metastasis and therapy resistance across carcinomas. Pharmacol. Ther..

[29] Grigore A., Jolly M.K., Jia D., Farach-Carson M., Levine H. (2016). Tumor Budding: The Name is EMT. Partial EMT. J. Clin. Med..

[30] Tripathi S., Levine H., Jolly M.K. (2020). The Physics of Cellular Decision-Making during Epithelial-Mesenchymal Transition. Annu. Rev. Biophys..

[31] Ghaleb A.M., Yang V.W. (2017). Krüppel-like factor 4 (KLF4): What we currently know. Gene.

[32] Nandan M.O., Yang V.W. (2009). The role of Krüppel-like factors in the reprogramming of somatic cells to induced pluripotent stem cells. Histol. Histopathol..

[33] Segre J.A., Bauer C., Fuchs E. (1999). Klf4 is a transcription factor required for establishing the barrier function of the skin. Nat. Genet..

[34] Cowan C.E., Kohler E.E., Dugan T.A., Mirza M.K., Malik A.B., Wary K.K. (2010). Krüppel-like factor-4 transcriptionally regulates VE-cadherin expression and endothelial barrier function. Circ. Res..

[35] Zhang P., Andrianakos R., Yang Y., Liu C., Lu W. (2010). Kruppel-like factor 4 (Klf4) prevents embryonic stem (ES) cell differentiation by regulating Nanog gene expression. J. Biol. Chem..

[36] Tiwari A., Loughner C.L., Swamynathan S., Swamynathan S.K. (2017). KLF4 plays an essential role in corneal epithelial homeostasis by promoting epithelial cell fate and suppressing epithelial-mesenchymal transition. Investig. Ophthalmol. Vis. Sci..

[37] Fujimoto S., Hayashi R., Hara S., Sasamoto Y., Harrington J., Tsujikawa M., Nishida K. (2019). KLF4 prevents epithelial to mesenchymal transition in human corneal epithelial cells via endogenous TGF-β2 suppression. Regen. Ther..

[38] Lin L., Han Q., Xiong Y., Li T., Liu Z., Xu H., Wu Y., Wang N., Liu X. (2017). Krüpple-like-factor 4 Attenuates Lung Fibrosis via Inhibiting Epithelial-mesenchymal Transition. Sci. Rep..

[39] Leng Z., Tao K., Xia Q., Tan J., Yue Z., Chen J., Xi H., Li J., Zheng H. (2013). Krüppel-Like Factor 4 Acts as an Oncogene in Colon Cancer Stem Cell-Enriched Spheroid Cells. PLoS ONE.

[40] Guan H., Xie L., Leithäuser F., Flossbach L., Möller P., Wirth T., Ushmorov A. (2010). KLF4 is a tumor suppressor in B-cell non-Hodgkin lymphoma and in classic Hodgkin lymphoma. Blood.

[41] Yu F., Li J., Chen H., Fu J., Ray S., Huang S., Zheng H., Ai W. (2011). Kruppel-like factor 4 (KLF4) is required for maintenance of breast cancer stem cells and for cell migration and invasion. Oncogene.

[42] Rowland B.D., Bernards R., Peeper D.S. (2005). The KLF4 tumour suppressor is a transcriptional repressor of p53 that acts as a context-dependent oncogene. Nat. Cell Biol..

[43] Yori J.L., Seachrist D.D., Johnson E., Lozada K.L., Schiemann W.P., Keri R.A. (2011). Krüppel-like Factor 4 Inhibits Tumorigenic Progression and Metastasis in a Mouse Model of Breast Cancer. Neoplasia.

[44] Li Z., Huang J., Shen S., Ding Z., Luo Q., Chen Z., Lu S. (2018). SIRT6 drives epithelial-to-mesenchymal transition and metastasis in non-small cell lung cancer via snail-dependent transrepression of KLF4. J. Exp. Clin. Cancer Res..

[45] Liu Y.-N., Abou-Kheir W., Yin J.J., Fang L., Hynes P., Casey O., Hu D., Wan Y., Seng V., Sheppard-Tillman H. (2012). Critical and Reciprocal Regulation of KLF4 and SLUG in Transforming Growth Factor-Initiated Prostate Cancer Epithelial-Mesenchymal Transition. Mol. Cell. Biol..

[46] Bolós V., Peinado H., Pérez-Moreno M.A., Fraga M.F., Esteller M., Cano A. (2003). The transcription factor Slug represses E-cadherin expression and induces epithelial to mesenchymal transitions: A comparison with Snail and E47 repressors. J. Cell Sci..

[47] Brabletz S., Brabletz T. (2010). The ZEB/miR-200 feedback loop—A motor of cellular plasticity in development and cancer?. EMBO Rep..

[48] Subbalakshmi A.R., Sahoo S., Biswas K., Jolly M.K. (2021). A computational systems biology approach identifies SLUG as a mediator of partial Epithelial-Mesenchymal Transition (EMT). Cells Tissues Organs.

[49] Duddu A.S., Sahoo S., Hati S., Jhunjhunwala S., Jolly M.K. (2020). Multi-stability in cellular differentiation enabled by a network of three mutually repressing master regulators. J. R. Soc. Interface.

[50] Dang D.T., Zhao W., Mahatan C.S., Geiman D.E., Yang V.W. (2002). Opposing effects of Krüppel-like factor 4 (gut-enriched Krüppel-like factor) and Krüpple-like factor 5 (intestinal-enriched Krüppel-like factor) on the promoter of the Krüppel-like factor 4 gene. Nucleic Acids Res..

[51] Jolly M.K., Preca B.-T., Tripathi S.C., Jia D., George J.T., Hanash S.M., Brabletz T., Stemmler M.P., Maurer J., Levine H. (2018). Interconnected feedback loops among ESRP1, HAS2, and CD44 regulate epithelial-mesenchymal plasticity in cancer. APL BioEng..

[52] Varma S., Cao Y., Tagne J.B., Lakshminarayanan M., Li J., Friedman T.B., Morell R.J., Warburton D., Kotton D.N., Ramirez M.I. (2012). The transcription factors grainyhead-like 2 and NK2-homeobox 1 form a regulatory loop that coordinates lung epithelial cell morphogenesis and differentiation. J. Biol. Chem..

[53] Farris J.C., Pifer P.M., Zheng L., Gottlieb E., Denvir J., Frisch S.M. (2016). Grainyhead-like 2 reverses the metabolic changes induced by the oncogenic epithelial-mesenchymal transition: Effects on anoikis. Mol. Cancer Res..

[54] Aue A., Hinze C., Walentin K., Ruffert J., Yurtdas Y., Werth M., Chen W., Rabien A., Kilic E., Schulzke J.D. (2015). A grainyhead-like 2/Ovo-like 2 pathway regulates renal epithelial barrier function and lumen expansion. J. Am. Soc. Nephrol..

[55] Huang B., Lu M., Jia D., Ben-Jacob E., Levine H., Onuchic J.N. (2017). Interrogating the topological robustness of gene regulatory circuits by randomization. PLoS Comput. Biol..

[56] Chakraborty P., George J.T., Tripathi S., Levine H., Jolly M.K. (2020). Comparative Study of Transcriptomics-Based Scoring Metrics for the Epithelial-Hybrid-Mesenchymal Spectrum. Front. Bioeng. Biotechnol..

[57] Chakraborty P., Chen E.L., McMullen I., Armstrong A.J., Jolly M.K., Somarelli J.A. (2021). Analysis of immune subtypes across the epithelial-mesenchymal plasticity spectrum. Comput. Struct. Biotechnol. J..

[58] Chauhan L., Ram U., Hari K., Jolly M.K. (2021). Topological signatures in regulatory network enable phenotypic heterogeneity in small cell lung cancer. Elife.

[59] Johansson J., Tabor V., Wikell A., Jalkanen S., Fuxe J. (2015). TGF-b1-Induced Epithelial-Mesenchymal Transition Promotes Monocyte/Macrophage Properties in Breast Cancer Cells. Front. Oncol..

[60] McGrail D.J., Mezencev R., Kieu Q.M.N., McDonald J.F., Dawson M.R. (2015). SNAIL-induced epithelial-to-mesenchymal transition produces concerted biophysical changes from altered cytoskeletal gene expression. FASEB J..

[61] Chung V.Y., Tan T.Z., Ye J., Huang R.-L., Lai H.-C., Kappei D., Wollmann H., Guccione E., Huang R.Y.-J. (2019). The role of GRHL2 and epigenetic remodeling in epithelial–mesenchymal plasticity in ovarian cancer cells. Commun. Biol..

[62] Hong D., Messier T.L., Tye C.E., Dobson J.R., Fritz A.J., Sikora K.R., Browne G., Stein J.L., Lian J.B., Stein G.S. (2017). Runx1 stabilizes the mammary epithelial cell phenotype and prevents epithelial to mesenchymal transition. Oncotarget.

[63] Tan T.Z., Miow Q.H., Miki Y., Noda T., Mori S., Huang R.Y.-J., Thiery J.P. (2014). Epithelial-mesenchymal transition spectrum quantification and its efficacy in deciphering survival and drug responses of cancer patients. EMBO Mol. Med..

[64] Xiao X., Tang W., Yuan Q., Peng L., Yu P. (2015). Epigenetic repression of Krüppel-like factor 4 through Dnmt1 contributes to EMT in renal fibrosis. Int. J. Mol. Med..

[65] Li H., Wang J., Xiao W., Xia D., Lang B., Yu G., Guo X., Guan W., Wang Z., Hu Z. (2013). Epigenetic alterations of krüppel-like factor 4 and its tumor suppressor function in renal cell carcinoma. Carcinogenesis.

[66] Jia W., Tripathi S., Chakraborty P., Chedere A., Rangarajan A., Levine H., Jolly M.K. (2020). Epigenetic feedback and stochastic partitioning during cell division can drive resistance to EMT. Oncotarget.

[67] Jia W., Deshmukh A., Mani S.A., Jolly M.K., Levine H. (2019). A possible role for epigenetic feedback regulation in the dynamics of the epithelial-mesenchymal transition (EMT). Phys. Biol..

[68] Miyamoto T., Furusawa C., Kaneko K. (2015). Pluripotency, Differentiation, and Reprogramming: A Gene Expression Dynamics Model with Epigenetic Feedback Regulation. PLoS Comput. Biol..

[69] Clarke C., Madden S., Doolan P., Aherne S., Joyce H., O’Driscoll L., Gallagher W., Hennessy B., Moriarty M., Crown J. (2013). Correlating transcriptional networks to breast cancer survival: A large-scale coexpression analysis. Carcinogenesis.

[70] Miller L., Smeds J., George J., Vega V., Vergara L., Ploner A., Pawitan Y., Hall P., Klaar S., Liu E. (2005). An expression signature for p53 status in human breast cancer predicts mutation status, transcriptional effects, and patient survival. Proc. Natl. Acad. Sci. USA.

[71] Bonome T., Levine D.A., Shih J., Randonovich M., Pise-Masison C.A., Bogomolniy F., Ozbun L., Brady J., Barrett J.C., Boyd J. (2008). A Gene Signature Predicting for Survival in Suboptimally DebulkedPatients with Ovarian Cancer. Cancer Res..

[72] Ferriss J.S., Kim Y., Duska L., Birrer M., Levine D.A., Moskaluk C., Theodorescu D., Lee J.K. (2012). Multi-Gene Expression Predictors of Single Drug Responses to Adjuvant Chemotherapy in Ovarian Carcinoma: Predicting Platinum Resistance. PLoS ONE.

[73] Rousseaux S., Debernardi A., Jacquiau B., Vitte A.-L., Vesin A., Nagy-Mignotte H., Moro-Sibilot D., Brichon P.-Y., Lantuejoul S., Hainaut P. (2013). Ectopic Activation of Germline and Placental Genes Identifies Aggressive Metastasis-Prone Lung Cancers. Sci. Transl. Med..

[74] Győrffy B., Surowiak P., Budczies J., Lánczky A. (2013). Online survival analysis software to assess the prognostic value of biomarkers using transcriptomic data in non-small-cell lung cancer. PLoS ONE.

[75] George J.T., Jolly M.K., Xu S., Somarelli J.A., Levine H. (2017). Survival outcomes in cancer patients predicted by a partial EMT gene expression scoring metric. Cancer Res..

[76] Chen X., Whitney E.M., Gao S.Y., Yang V.W. (2003). Transcriptional profiling of Krüppel-like factor 4 reveals a function in cell cycle regulation and epithelial differentiation. J. Mol. Biol..

[77] Li X., Zhao Z., Zhang X., Yang S., Lin X., Yang X., Lin X., Shi J., Wang S., Zhao W. (2017). Klf 4 reduces stemness phenotype triggers mesenchymal-epithelial transition (MET)-like molecular changes, and prevents tumor progression in nasopharygeal carcinoma. Oncotarget.

[78] Yori J.L., Johnson E., Zhou G., Jain M.K., Keri R.A. (2010). Krüppel-like factor 4 inhibits epithelial-to-mesenchymal transition through regulation of E-cadherin gene expression. J. Biol. Chem..

[79] Chen Z., Wang Y., Liu W., Zhao G., Lee S., Balogh A., Zou Y., Guo Y., Zhang Z., Gu W. (2014). Doxycycline inducible Kruppel-like factor 4 lentiviral vector mediates mesenchymal to epithelial transition in ovarian cancer cells. PLoS ONE.

[80] Xiong X., Schober M., Tassone E., Khodadadi-Jamayran A., Sastre-Perona A., Zhou H., Tsirigos A., Shen S., Chang M., Melamed J. (2018). KLF4, A Gene Regulating Prostate Stem Cell Homeostasis, Is a Barrier to Malignant Progression and Predictor of Good Prognosis in Prostate Cancer. Cell Rep..

[81] Zhu Z., Yu Z., Wang J., Zhou L., Zhang J., Yao B., Dou J., Qiu Z., Huang C. (2018). Krüppel-Like Factor 4 Inhibits Pancreatic Cancer Epithelial-to-Mesenchymal Transition and Metastasis by Down-Regulating Caveolin-1 Expression. Cell. Physiol. Biochem..

[82] Jolly M.K., Tripathi S.C., Somarelli J.A., Hanash S.M., Levine H. (2017). Epithelial-mesenchymal plasticity: How have quantitative mathematical models helped improve our understanding?. Mol. Oncol..

[83] Yu F., Shi Y., Wang J., Li J., Fan D., Ai W. (2013). Deficiency of Kruppel-like factor KLF4 in mammary tumor cells inhibits tumor growth and pulmonary metastasis and is accompanied by compromised recruitment of myeloid-derived suppressor cells. Int. J. Cancer.

[84] Karagonlar Z.F., Akbari S., Karabicici M., Sahin E., Avci S.T., Ersoy N., Ates K.E., Balli T., Karacicek B., Kaplan K.N. (2020). A Novel Function for KLF4 in Modulating the De-differentiation of EpCAM-/CD133- nonStem Cells into EpCAM+/CD133+ Liver Cancer Stem Cells in HCC Cell Line HuH7. Cells.

[85] Li Y., Xian M., Yang B., Ying M., He Q. (2017). Inhibition of KLF4 by Statins Reverses Adriamycin-Induced Metastasis and Cancer Stemness in Osteosarcoma Cells. Stem Cell Rep..

[86] Qi X.-T., Li Y.-L., Zhang Y.-Q., Xu T., Lu B., Fang L., Gao J.-Q., Yu L.-S., Zhu D.-F., Yang B. (2019). KLF4 functions as an oncogene in promoting cancer stem cell-like characteristics in osteosarcoma cells. Acta Pharmacol. Sin..

[87] Martins-Neve S.R., Corver W.E., Paiva-Oliviera D.I., van den Akker B.E.W.M., Briaire-de-Bruijn I.H., Bovee J.V.M.G., Gomes C.M.F., Cleton-Jansen A.-M. (2016). Osteosarcoma Stem Cells Have Active Wnt/β-catenin and Overexpress SOX2 and KLF4. J. Cell. Physiol..

[88] Liu S., Cong Y., Wang D., Sun Y., Deng L., Liu Y., Martin-Trevino R., Shang L., McDermott S.P., Landis M.D. (2014). Breast cancer stem cells transition between epithelial and mesenchymal states reflective of their normal counterparts. Stem Cell Rep..

[89] Luo M., Shang L., Brooks M.D., Jiagge E., Zhu Y., Buschhaus J., Conley S., Fath M.A., Davis A., Gheordunescu E. (2018). Targeting Breast Cancer Stem Cell State Equilibrium through Modulation of Redox Signaling. Cell Metab..

[90] Nishimura K., Aizawa S., Nugroho F.L., Shiomitsu E., Tran Y.T.H., Bui P.L., Borisova E., Sakuragi Y., Takada H., Kurisaki A. (2017). A Role for KLF4 in Promoting the Metabolic Shift via TCL1 during Induced Pluripotent Stem Cell Generation. Stem Cell Rep..

[91] Moon J.S., Kim H.E., Koh E., Park S.H., Jin W.J., Park B.W., Park S.W., Kim K.S. (2011). Krüppel-like factor 4 (KLF4) activates the transcription of the gene for the platelet isoform of phosphofructokinase (PFKP) in breast cancer. J. Biol. Chem..

[92] Tiwari N., Meyer-Schaller N., Arnold P., Antoniadis H., Pachkov M., van Nimwegen E., Christofori G. (2013). Klf4 Is a Transcriptional Regulator of Genes Critical for EMT, Including Jnk1 (Mapk8). PLoS ONE.

[93] Yan Y., Li Z., Kong X., Jia Z., Zuo X., Gagea M., Huang S., Wei D., Xie K. (2016). KLF4-mediated suppression of CD44 signaling negatively impacts pancreatic cancer stemness and metastasis. Cancer Res..

[94] Murgai M., Ju W., Eason M., Kline J., Beury D.W., Kaczanowska S., Miettinen M.M., Kruhlak M., Lei H., Shern J.F. (2017). KLF4-dependent perivascular cell plasticity mediates pre-metastatic niche formation and metastasis. Nat. Med..

[95] Zhou Z., Feng Z., Hu D., Yang P., Gur M., Bahar I., Cristofanilli M., Gradishar W.J., Xie X.-q., Wan Y. (2019). A novel small-molecule antagonizes PRMT5-mediated KLF4 methylation for targeted therapy. EBioMedicine.

[96] Cercek A., Wheler J., Murray P.E., Zhou S., Saltz L. (2015). Phase 1 study of APTO-253 HCl, an inducer of KLF4, in patients with advanced or metastatic solid tumors. Investig. New Drugs.

[97] Karacosta L.G., Anchang B., Ignatiadis N., Kimmey S.C., Benson J.A., Shrager J.B., Tibshirani R., Bendall S.C., Plevritis S.K. (2019). Mapping Lung Cancer Epithelial-Mesenchymal Transition States and Trajectories with Single-Cell Resolution. Nat. Commun..

[98] Tripathi S., Chakraborty P., Levine H., Jolly M.K. (2020). A mechanism for epithelial-mesenchymal heterogeneity in a population of cancer cells. PLoS Comput. Biol..

[99] Dhooge A., Govaerts W., Kuznetsov Y.A. (2003). MATCONT: A MATLAB Package for Numerical Bifurcation Analysis of ODEs. ACM Trans. Math. Softw..

[100] Davis S., Meltzer P.S. (2007). GEOquery: A bridge between the Gene Expression Omnibus (GEO) and BioConductor. Bioinformatics.

[101] Wang Z., Wu X., Wang Y. (2018). A framework for analyzing DNA methylation data from Illumina Infinium HumanMethylation450 BeadChip. BMC Bioinform..

[102] Mandal S., Tejaswi T., Janivara R., Srikrishnan S., Thakur P., Sahoo S., Chakraborty P., Sohal S.S., Levine H., George J.T. (2021). Transcriptomic-based quantification of the epithelial-hybrid-mesenchymal spectrum across biological contexts. bioRxiv.

