# Supplementary material for: KLF4 Induces Mesenchymal–Epithelial Transition (MET) by Suppressing Multiple EMT-Inducing Transcription Factors
Source: Cancers (Basel). 2021 Oct 13;13(20):5135. doi: 10.3390/cancers13205135 (PMC8533753; doi:10.3390/cancers13205135)
Supplement: Supplementary file 1 [file cancers-13-05135-s001.zip › cancers-1377842-supplementary.pdf]

File S1: KLF4_SI

The dynamics of the molecular species of the EMT regulatory circuit (miR-200, Snail, Zeb, Slug) and KLF4 (shown in Fig 1A) is described using coupled ordinary differential equations.

$$\frac{d\mu_{200}}{dt} = g_{\mu_{200}} H^s(Z,\lambda_{Z,\mu_{200}}) H^s(S,\lambda_{S,\mu_{200}}) H^s(Sl,\lambda_{Sl,\mu_{200}}) - m_Z Y_\mu(\mu_{200}) - m_{Sl} Y_\mu(\mu_{200}) - k_{\mu_{200}} \mu_{200}$$

$$\frac{dm_Z}{dt} = g_{m_Z} H^s(Z,\lambda_{Z,m_Z}) H^s(S,\lambda_{S,m_Z}) - m_Z Y_Z(\mu_{200}) - k_{m_Z} m_Z$$

$$\frac{dZ}{dt} = g_Z m_Z L(\mu_{200}) - k_Z Z$$

$$\frac{dS}{dt} = g_S H^s(I,\lambda_{I,S}) H^s(Sl,\lambda_{Sl,S}) H^s(S,\lambda_{S,S}) H^s(K,\lambda_{K,m_S}) - k_S S$$

$$\frac{dm_{Sl}}{dt} = g_{m_{Sl}} H^s(S,\lambda_{S,m_{Sl}}) H^s(K,\lambda_{K,m_{Sl}}) - m_{Sl} Y_Z(\mu_{200}) - k_{m_{Sl}} m_{Sl}$$

$$\frac{dSl}{dt} = g_{Sl} m_{Sl} L(\mu_{200}) - k_{Sl} Sl$$

$$\frac{dK}{dt} = g_K H^s(K,\lambda_{K,K}) H^s(Sl,\lambda_{Sl,S}) H^s(S,\lambda_{S,S}) - k_S S$$

where g_X is the corresponding innate production rate and k_X is the innate degradation rate.

$m_z L(\mu_{200})$ is the net translation rate, $m_z Y_m(\mu_{200})$ is the total ZEB mRNA active degradation rate and $m_z Y_\mu(\mu_{200})$ is the total miR active degradation rate. H^S is the shifted Hill function, defined as

$H^S(B,\lambda) = H^-(B) + \lambda H^+(B)$,

$H^-(B) = 1/\,[1+(B/B_0)^{nB}]$,

$H^+(B) = 1 - H^-(B)$,

λ is the fold change from the basal synthesis rate due to protein B. $\lambda > 1$ for activators, while $\lambda < 1$ for inhibitors.

λ<1 for inhibitors.

Parameter Estimation:

The model parameters were adopted from previously published literature for the molecular species of the core circuit (I, miR-200, Snail, Zeb, Slug) and KLF4 interactions, as given below:

Parameter	Value	Reference
$g\mu 200$ (Molecules/Hour)	2.1K	[1]
gmZ (Molecules/Hour)	11	[1]
$Z^0\mu_{200}$ (Molecules)	220K	[1]
$Z^0 m_Z$ (Molecules)	25K	[1]
$n_{Z,\mu_{200}}$	3	[1]
n_{Z,m_Z}	2	[1]
$n_{\mu_{200}}$	6	[1]
$n_{S,\mu_{200}}$	2	[1]
n_{S,m_Z}	2	[1]
$\lambda_{Z,\mu_{200}}$	0.1	[1]

λ_{Z,m_Z}	7.5	[1]
$\lambda_{S,\mu_{200}}$	0.1	[1]
λ_{S,m_Z}	10	[1]
$k_{\mu_{200}}$(Hour^{-1})	0.05	[1]
k_{m_Z}(Hour^{-1})	0.5	[1]
k_Z(Hour^{-1})	0.1	[1]
g_Z(Hour^{-1})	0.1K	[1]
$S^0_{\mu_{200}}$(Molecules)	180K	[1]
$S^0_{m_Z}$(Molecules)	180K	[1]
μ^0_{200}(Molecules)	10K	[1]
g_S	18000	[1]
k_S	0.125	[1]
g_{Sl}	50000	Estimated
k_{Sl}	0.1155	[2]
$g_{m_{Sl}}$	90	Estimated
$k_{m_{Sl}}$	0.5	Estimated
$\lambda_{Sl,\mu_{200}}$	0.4	[3]
$\lambda_{Sl,S}$	0.5	[4]
$\lambda_{S,S}$	0.4	[5]
$\lambda_{S,m_{Sl}}$	0.5	[4]
$n_{Sl,\mu_{200}}$	1	[3]
$n_{Sl,S}$	3	[6]
$n_{S,S}$	5	[6]
$n_{S,m_{Sl}}$	1	[6]
$Sl^0_{\mu_{200}}$	220000	Estimated
Sl^0_S	225000	Estimated
S^0_S	300000	Estimated
$S^0_{m_{Sl}}$	180000	Estimated
nIs	2	[7]
I^0S	100000	[7]
$\lambda_{I,S}$	3	[7]
g_K	50000	
k_K	0.1732	[8]
$\lambda_{K,S}$	0.5	[9]
$\lambda_{K,m_{Sl}}$	0.25	[10]
$\lambda_{S,K}$	0.25	[11]
$\lambda_{Sl,K}$	0.5	[10]
$n_{K,S}$	2	[9]
$n_{K,m_{Sl}}$	2	[10]
$n_{S,K}$	2	estimated
$n_{Sl,K}$	4	[10]
$\lambda_{K,K}$	2	[12]
$n_{K,K}$	3	[13]
S^0_K	180000	Estimated
$K^0_{m_{Sl}}$	300000	Estimated
Sl^0_K	225000	Estimated
K^0_K	250000	Estimated
K^0_S	275000	Estimated

Datasets Used in Kaplan-Meier analysis:

Dataset	n(High)	n(Low)
GSE42568 (Breast Cancer Sample)	52	52
GSE45255 (Breast Cancer Sample)	67	67
GSE30219 (Lung Cancer Sample)	147	146
CaArray (Lung Cancer Sample)	234	234
GSE30161 (Ovarian Cancer Sample)	28	30
GSE26712 (Ovarian Cancer Sample)	60	124

Supplementary figures:

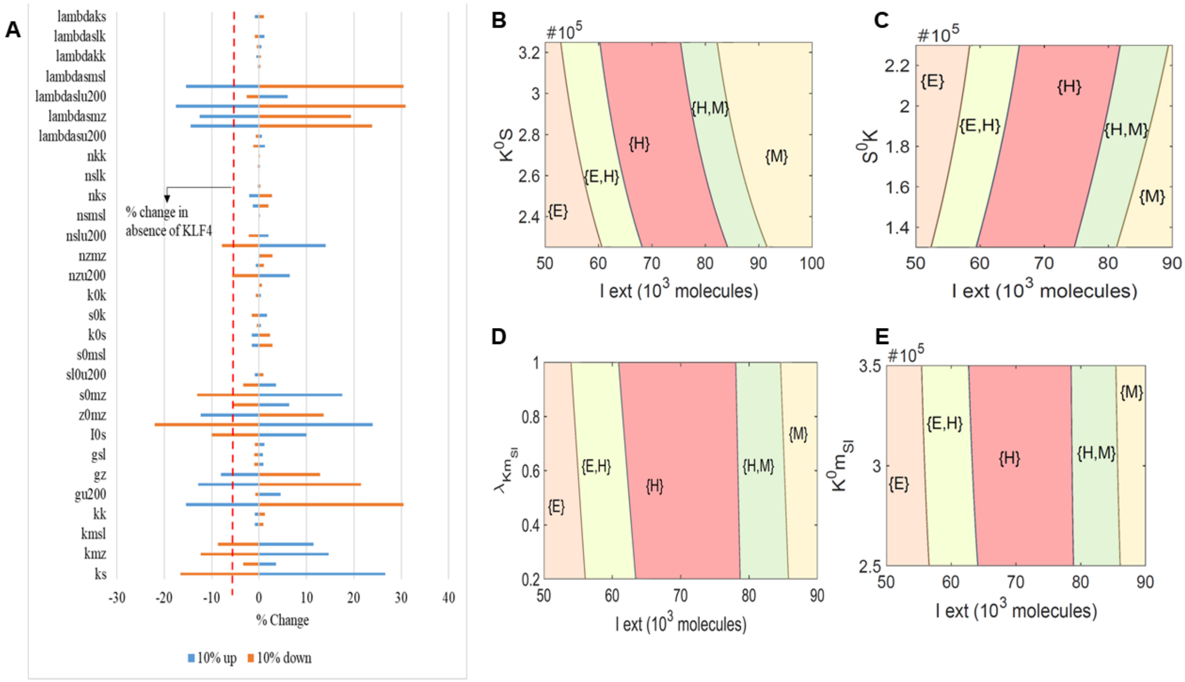

Figure S1: A) Sensitivity analysis indicating percent change in the interval of external EMT signal levels for stable hybrid E/M region, when corresponding parameter values are varied by ±10%. The red dotted line indicates the percent change in the stable hybrid region in the absence of KLF4 (core network) when compared to the coupled network with KLF4. ***B)*** Phase diagrams for the KLF4 network driven by an external signal (I) for varying threshold levels of KLF4 needed for repression on SNAIL. ***C)*** Phase diagrams for the KLF4 network driven by an external signal (I) for varying threshold levels of SNAIL needed for repression on KLF4. ***D)*** Phase diagrams for the KLF4 network driven by an external signal (I) for varying strength of repression on SLUG mRNA by KLF4. ***E)*** Phase diagrams for the KLF4 network driven by an external signal (I) for varying threshold levels of KLF4 needed for repression on SLUG.

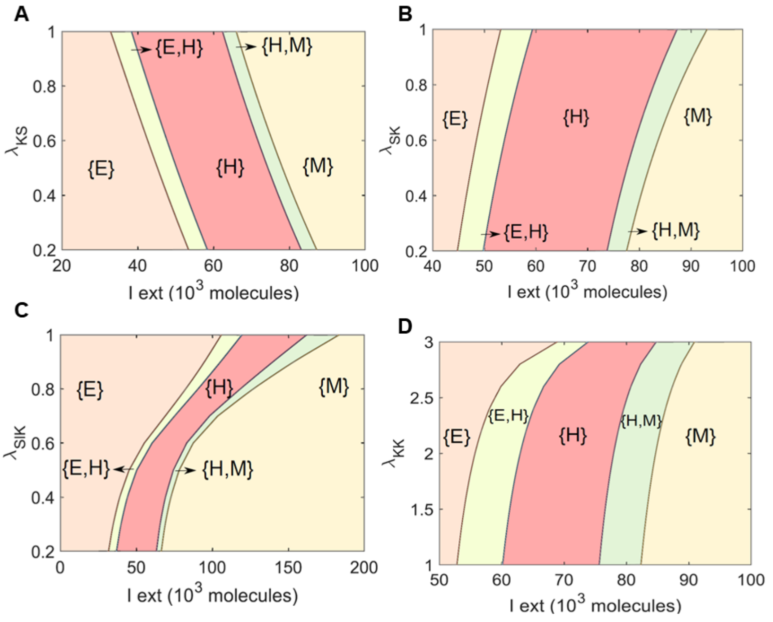

Figure S2: Effect of SLUG self-activation A) Phase diagrams for the KLF4 network with SLUG self-activation driven by an external signal (I) for varying strength of repression on SNAIL by KLF4. ***B)*** Phase diagrams for the KLF4 network with SLUG self-activation driven by an external signal (I) for varying strength of repression on KLF4 by SNAIL. ***C)*** Phase diagrams for the KLF4 network with SLUG self-activation driven by an external signal (I) for varying strength of repression on SLUG by KLF4. ***D)*** Phase diagrams for the KLF4 network with SLUG self-activation driven by an external signal (I) for varying strength of KLF4 self-activation.

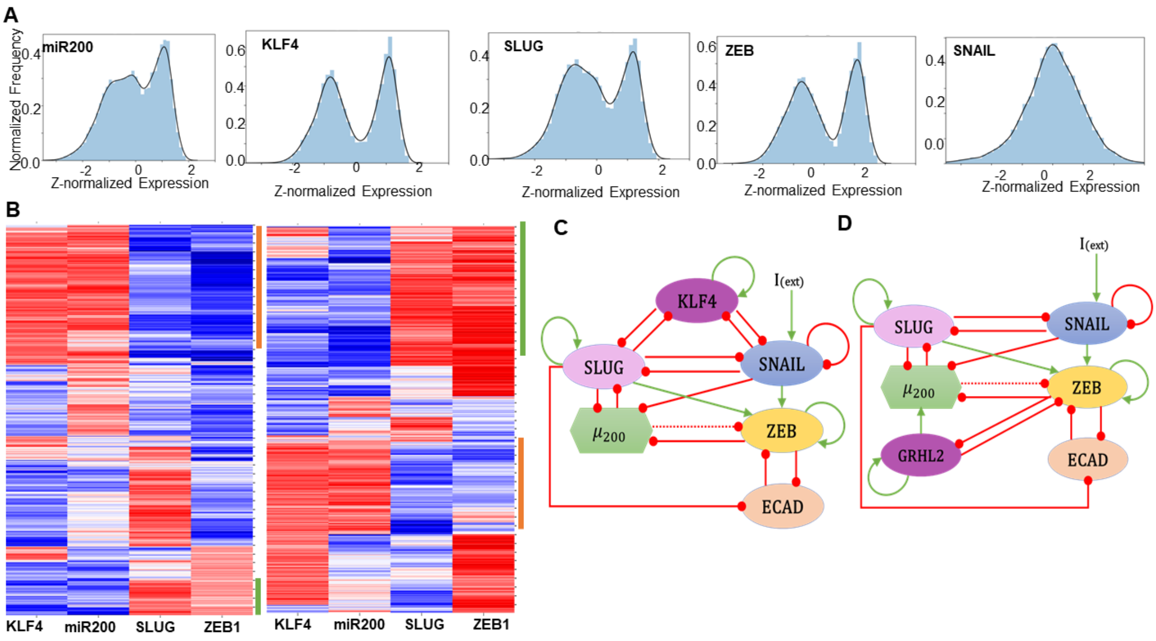

Figure S3: RACIPE analysis of KLF4-EMT circuit. A) Normalized histograms of the expression levels of the nodes in the GRN. ***B)*** Heatmap showing the steady-state expression levels of a single replicate involving ZEB down-expression (ZEB DE) on the simulated GRN ***(Left panel)***. Heatmap showing the steady-state expression levels of a single replicate involving ZEB over-expression (ZEB OE) on the simulated GRN ***(Right panel)***. Green represents mesenchymal phenotype and orange represents epithelial phenotype. ***C)*** Extended KLF4 gene regulatory network ***D)*** GRN containing GRHL2 used for RACIPE simulations

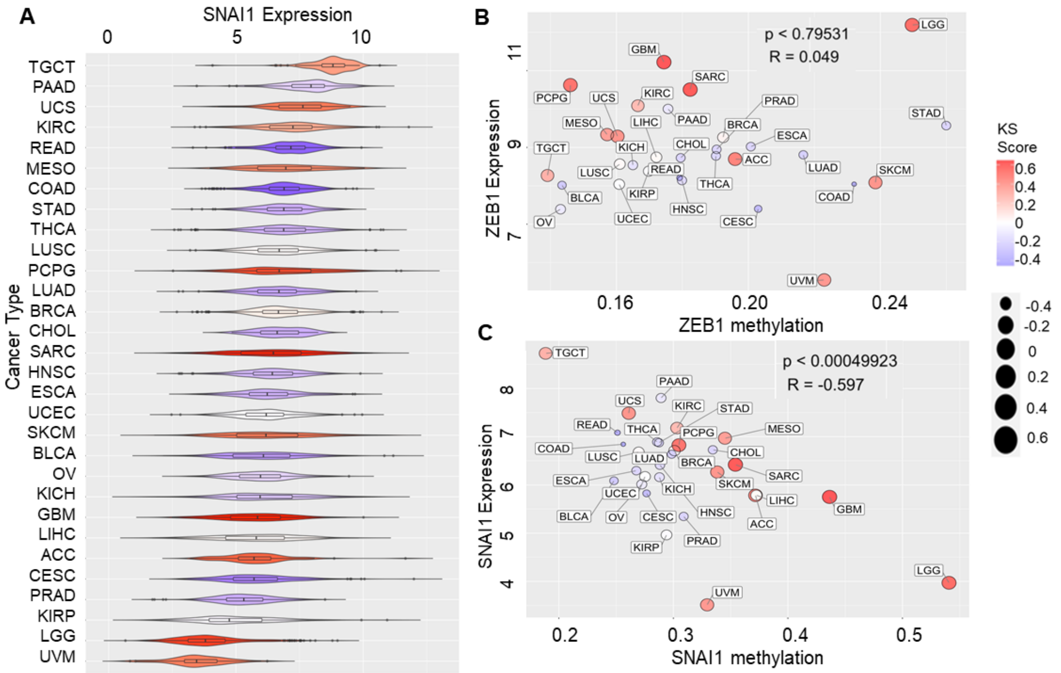

Figure S4: A) SNAI1 expression in TCGA cancers in relation to KS score ***B)*** ZEB1 expression in TCGA cancers in relation to its methylation status ***C)*** SNAI1 expression in TCGA cancers in relation to its methylation status

References:

1. Lu, M.; Jolly, M. K.; Levine, H.; Onuchic, J. N.; Ben-Jacob, E. MicroRNA-based regulation of epithelial-hybrid-mesenchymal fate determination. *Proc. Natl. Acad. Sci.* **2013**, *110*, 18144–18149.

2. Molina-Ortiz, P.; Villarejo, A.; MacPherson, M.; Santos, V.; Montes, A.; Souchelnytskyi, S.; Portillo, F.; Cano, A. Characterization of the SNAG and SLUG domains of Snail2 in the repression of E-cadherin and EMT induction: Modulation by serine 4 phosphorylation. *PLoS One* **2012**, *7*, e36132.

3. Liu, Y. N.; Yin, J. J.; Abou-Kheir, W.; Hynes, P. G.; Casey, O. M.; Fang, L.; Yi, M.; Stephens, R. M.; Seng, V.; Sheppard-Tillman, H.; Martin, P.; Kelly, K. MiR-1 and miR-200 inhibit EMT via Slug-dependent and tumorigenesis via Slug-independent mechanisms. *Oncogene* **2013**, *32*, 296–306.

4. Nakamura, R.; Ishii, H.; Endo, K.; Hotta, A.; Fujii, E.; Miyazawa, K.; Saitoh, M. Reciprocal expression of slug and snail in human oral cancer cells. *PLoS One* **2018**, *13*, e0199442.

5. Peiró, S.; Escrivà, M.; Puig, I.; Barberà, M. J.; Dave, N.; Herranz, N.; Larriba, M. J.; Takkunen, M.; Francí, C.; Muñoz, A.; Virtanen, I.; Baulida, J.; García de Herreros, A. Snail1 transcriptional repressor binds to its own promoter and controls its expression. *Nucleic Acids Res.* **2006**, *34*, 2077–2084.

6. Chen, Y.; Gridley, T. The SNAI1 and SNAI2 proteins occupy their own and each other's promoter during chondrogenesis. *Biochem. Biophys. Res. Commun.* **2013**, *435*, 356–360.

7. Jolly, M. K.; Boareto, M.; Debeb, B. G.; Aceto, N.; Farach-Carson, M. C.; Woodward, W. A.; Levine, H. Inflammatory Breast Cancer: a model for investigating cluster-based dissemination. *NPJ Breast Cancer* **2017**, *3*, 21.

8. Gamper, A. M.; Qiao, X.; Kim, J.; Zhang, L.; De Simone, M. C.; Rathmell, W. K.; Wan, Y. Regulation of KLF4 Turnover Reveals an Unexpected Tissue-Specific Role of pVHL in Tumorigenesis. *Mol. Cell* **2012**, *45*, 233–243.

9. Yori, J. L.; Seachrist, D. D.; Johnson, E.; Lozada, K. L.; Schiemann, W. P.; Keri, R. A. Krüppel-like Factor 4 Inhibits Tumorigenic Progression and Metastasis in a Mouse Model of Breast Cancer. *Neoplasia* **2011**, *13*, 601–610.

10. Liu, Y.-N.; Abou-Kheir, W.; Yin, J. J.; Fang, L.; Hynes, P.; Casey, O.; Hu, D.; Wan, Y.; Seng, V.; Sheppard-Tillman, H.; Martin, P.; Kelly, K. Critical and Reciprocal Regulation of KLF4 and SLUG in Transforming Growth Factor -Initiated Prostate Cancer Epithelial-Mesenchymal Transition. *Mol. Cell. Biol.* **2012**, *32*, 941–953.

11. Li, Z.; Huang, J.; Shen, S.; Ding, Z.; Luo, Q.; Chen, Z.; Lu, S. SIRT6 drives epithelial-to-mesenchymal transition and metastasis in non-small cell lung cancer via snail-dependent transrepression of KLF4. *J. Exp. Clin. Cancer Res.* **2018**, *37*, 323.

12. Dang, D. T. Opposing effects of Kruppel-like factor 4 (gut-enriched Kruppel-like factor) and Kruppel-like factor 5 (intestinal-enriched Kruppel-like factor) on the promoter of the Kruppel-like factor 4 gene. *Nucleic Acids Res.* **2002**, *30*, 2736–2741.

13. Mahatan, C. S.; Kaestner, K. H.; Geiman, D. E.; Yang, V. W. Characterization of the structure and regulation of the murine gene encoding gut-enriched Krüppel-like factor (Krüppel-like factor 4). *Nucleic Acids Res.* **1999**, *27*, 4562–4569.
